# Mechanical circulatory support reduces renal sympathetic nerve activity in an ovine model of acute myocardial infarction

**DOI:** 10.1007/s10286-024-01086-5

**Published:** 2024-11-27

**Authors:** Tania Warnakulasuriya, Bindu George, Nigel Lever, Rohit Ramchandra

**Affiliations:** 1 Manaaki Manawa – The Centre for Heart Research and the Department of Physiology, University of Auckland, New Zealand, Auckland, New Zealand; 2 Department of Physiology, Faculty of Medicine, University of Kelaniya, Kelaniya, Sri Lanka; 3 Auckland District Health Board: Te Whatu Ora Health New Zealand Te Toka Tumai Auckland, Auckland, New Zealand

**Keywords:** LVAD, RSNA, Acute myocardial infarction, Mechanical circulatory support

## Abstract

**Purpose:**

The use of circulatory assist devices has been shown to improve glomerular filtration rate and reduce the incidence of acute kidney injury in patients following acute cardiac pathology. However, the mechanisms of improvement in kidney function are not clear. We tested the hypothesis that mechanical circulatory support would result in a decrease in directly recorded renal sympathetic nerve activity (RSNA) and mediate the improvement in renal blood flow (RBF) in a setting of acute myocardial infarction (AMI)-induced left ventricular systolic dysfunction.

**Methods:**

An anaesthetized ovine model was used to induce AMI (*n* = 8) using injections of microspheres into the left coronary artery in one group. The second group did not undergo embolization (*n* = 6). The effects of mechanical circulatory support using the Impella CP on directly recorded renal sympathetic nerve activity were examined in these two groups of animals.

**Results:**

Injection of microspheres resulted in a drop in mean arterial pressure (MAP) of 21 ± 4 mmHg compared to baseline values (*p* < 0.05; *n* = 8). This was associated with a 67% increase in renal sympathetic nerve activity (RSNA; from 16 ± 5 to 21 ± 5 spikes/s; *p* < 0.05; *n* = 7). Impella CP support significantly increased MAP by 13 ± 1.5 mmHg at pump level 8 (*p* < 0.05) in the AMI group. Incremental pump support resulted in a significant decrease in RSNA (*p* < 0.05) in both groups. At pump level P8 in the AMI group, RSNA was decreased by 21 ± 5.5% compared to pump level P0 when the pump was not on.

**Conclusion:**

Our data indicate that the improvement in kidney function following mechanical circulatory support may be mediated in part by renal sympathoinhibition.

## Introduction

Acute myocardial ischaemia (AMI) continues to be a leading cause of mortality worldwide, causing 8.9 million deaths globally in 2019 [1]. Ventricular arrhythmias [2] and acute myocardial ischaemia-induced cardiogenic shock (AMI-CS) remain responsible for the high mortality rate even with advanced pharmacological and non-pharmacological management [3]. Left ventricular assist devices (LVADs) are gaining momentum as a left ventricular unloading therapy in the management of patients with AMI and with left ventricular dysfunction/pre-shock [4], cardiogenic shock and severe heart failure [5, 6].

AMI with left ventricular systolic dysfunction (LVSD) and hypotension is associated with decreased perfusion of many organs including the kidney [7, 8]. The incidence of acute kidney injury (AKI) in patients with AMI is high, and mortality is elevated in patients with these dual insults [9]. Unloading of the left ventricle using a mechanical circulatory assist pump (Impella, Abiomed Europe GmbH, Aachen, Germany) has been shown to improve glomerular filtration rate (GFR) [10, 11] and thereby reduce the incidence of AKI in patients with cardiac pathology. The improvement in GFR with the Impella pump is also associated with a reduction in the renal resistive index (RRI) [12] and has been hypothesized to mediate the decreased incidence of AKI in these patients. Furthermore, the use of assist devices reduce the need for vasoconstrictor medication, which further helps to prevent nephrotoxicity by these pharmacological agents [13]. What remains unclear is whether the improvement in blood flow to the kidney is mediated solely by the improved cardiac output or if other factors play a role.

In this context, the kidneys are innervated by a dense network of sympathetic nerves that play an important role in modulating blood flow and sodium excretion, as well as control of renin secretion [14]. Whether LVADs alter sympathetic nerve activity to the kidney remains unknown. We hypothesized that use of the Impella pump would result in a decrease in renal sympathetic nerve activity (RSNA) and mediate the improvement in renal blood flow (RBF). We tested this hypothesis using direct recordings of RSNA in a large animal model of AMI.

## Methods

### Animals

**Fig. 1:**
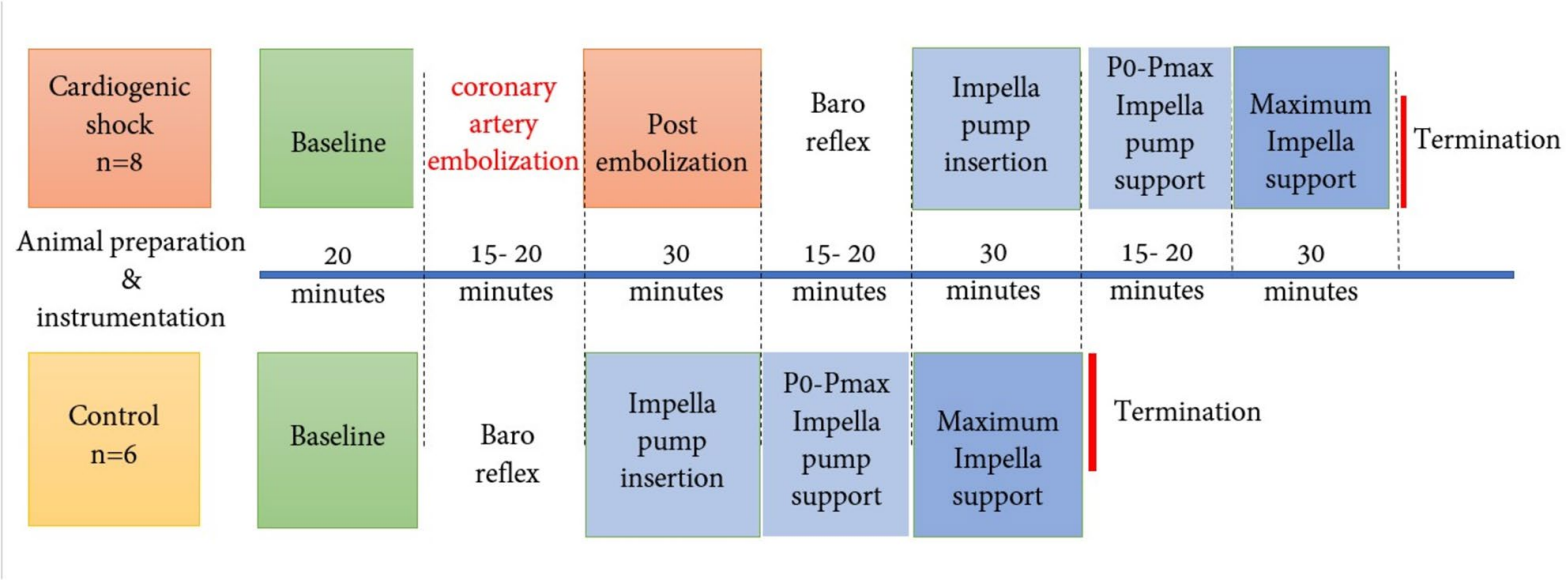
Experimental protocol illustrating the procedures performed on the acute myocardial infarction and control groups

This study was conducted on adult female Romney sheep (total of *n* = 18 sheep) with a mean weight of 58 ± 6.6 kg (range 48–69 kg). All experiments were reviewed by the Animal Ethics Committee of the University of Auckland and approved for use of the animals for this research (University of Auckland Animal Ethics Committee; Study, AEC24524). The number of animals used was based on power calculations done prior to the start of experiments and are noted throughout the manuscript. These studies were only conducted in ewes. The sheep were fasted for 24 h before the acute experiment under anaesthesia. The study groups comprised an AMI group (*n* = 10), and these were compared to a control group (*n* = 8), so a total of *n* = 18 animals were used. Animals were randomly allocated to each group. The control group underwent induction of anaesthesia, surgical instrumentation, Impella pump insertion and the study protocol as outlined in Fig. 1. The study group underwent induction of AMI by left coronary artery embolization under fluoroscopy before the Impella pump insertion.

### Induction of anaesthesia

Induction of anaesthesia was performed using 2% propofol (Diprivan) (5 mg/kg intravenously [i.v.], AstraZeneca, Australia) and maintained with a 2% isoflurane-air-O_2_ mixture. The animals were intubated for mechanical ventilation. An absence of the corneal reflex and an absence of a withdrawal response to a noxious pinch were monitored to determine the depth of anaesthesia. Pulse oximeter measurements were monitored to establish haemodynamic stability. An experienced animal handler/anaesthetic technician carried out the above procedure. Fluid was given intravenously (500 ml/hour) to maintain volume loss during the experiment. Heparin (150 IU/kg) was given as a bolus following vascular access before instrumentation and hourly (5000 IU) while on the Impella pump to avoid clotting. Sheep were placed on their right side and instrumentation was carried out under aseptic conditions.

### Surgical instrumentation

An incision was made in the left flank. The left renal artery and renal nerve were exposed using methods routine in the lab [15, 16]. A size 6 transonic flow probe was placed on the left renal artery (6PS, Transonic Systems, USA) to measure RBF. RSNA was recorded between a pair of custom-made stainless steel electrodes as described previously [17], and the baseline RSNA was established. The signal was amplified (×20,000) and filtered (bandpass 400–1200 Hz).

Following this, the sheep were placed in a supine cradle position. A 10-cm incision was made on the left side of the neck. A cannula was inserted into the left jugular vein for venous infusion and venous blood sampling. The catheter was secured with a purse-string suture (Filasilk, 3.0 non-absorbable braided silk suture) to maintain blood flow through the vessel. To record blood pressure (BP), a solid-state pressure catheter (Millar Inc., TX, USA) was inserted into the right femoral artery through an 8F introducer using a modified Seldinger technique. Four limb electrodes were inserted to record the electrocardiogram (ECG).

### Induction of AMI with left ventricular systolic dysfunction

**Fig. 2:**
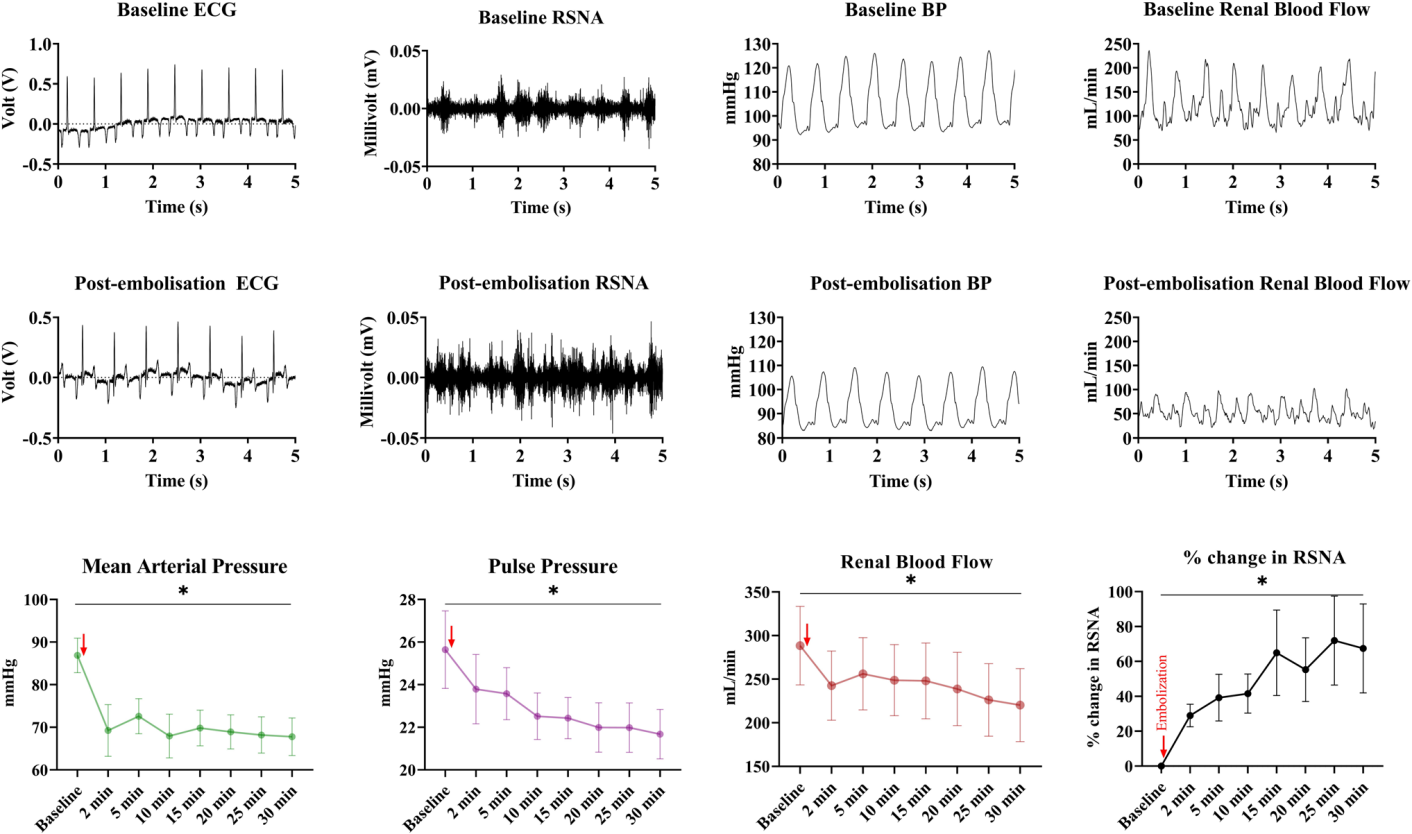
Representative raw data traces of ECG, renal sympathetic nerve activity (RSNA), blood pressure (BP) and renal blood flow changes in one animal before (first row) and 30 min after (second row) coronary artery embolization. The third row denotes the changes to mean arterial pressure, pulse pressure (**n** = 8) and renal blood flow and % change in renal sympathetic nerve activity (RSNA) from baseline (**n** = 7) before and serially after embolization of the left coronary artery. *p < 0.05 (one-way ANOVA). Embolization of the left coronary artery is indicated by the red arrow

The right femoral artery was accessed percutaneously with an 8F (CORDIS^®^, USA) sheath. The left main coronary artery was then cannulated using an 8F AL2 (CORDIS^®^, USA) guide catheter under fluoroscopic guidance. Following this, 1.5–2.0 ml polystyrene latex microspheres (45 microns; 1.3 ml, Polysciences, Warrington, PA, USA) was infused into the catheter. A change in the ST segment (elevation or depression) and the T wave (inversion) on ECG were noted as an indication of a successful embolization resulting in myocardial ischaemia (Fig. 2). If the drop in BP was not sustained, a smaller dose of microspheres were infused into the coronary arteries until there was a sustained reduction in BP. The BP, RBF and RSNA were recorded for the next 30 min. A mean arterial pressure reduction of ~15 mmHg was noted as successful induction of LVSD.

### Insertion of Impella CP

The implantation of the Impella CP (Abiomed Europe GmbH, Aachen, Germany) was carried out according to the manufacturer’s recommendations following training. Purge fluid was prepared using 5% glucose with 20 IU/ml of heparin. Vascular access was obtained by an arterial cut-down procedure of the common carotid artery. A 6Fr, 60-cm pigtail angiographic catheter with a guide wire (0.035-inch, polytetrafluoroethylene [PTFE]-coated, standard J tip) was inserted into the common carotid artery and guided into the left ventricle through the aortic valve with echocardiographic guidance. The 0.035-inch guidewire was taken out and a softer 0.018-inch placement guidewire was inserted into the angiographic catheter. Once the ventricular placement of the soft guidewire was confirmed by echocardiography, the angiographic pigtail catheter was removed with echocardiographic monitoring.

The 0.018-inch guidewire was loaded into the Impella CP pump which was advanced along the guidewire into the left ventricle. The guidewire was removed and the Impella inlet position was adjusted to be in the mid-ventricular cavity and the outlet in the ascending aorta. The Impella controller recorded the pressure waveform, and the pressure wave was confirmed to be of aortic origin. The pump was started at level 1 (P1). Colour Doppler was used to confirm the inflow and outflow areas, and the position was adjusted as required. Once the positioning was satisfactory, the flow rate was increased every 3 min until level 8 or suction was noted. If suction was noted at a pump level, the pump level was reduced and positioning was re-adjusted, and the higher level repeated. P0 was recorded with the pump placed in the ventricle.

The pump support levels were randomly altered every 2 min at different speeds. Changes to arterial pressure, RBF and RSNA were recorded at each pump level.

### Baroreflex assessment

Changes in BP and RSNA were recorded in response to intravenous infusion of phenylephrine. This was done after embolization in the AMI group but before the Impella pump insertion in order to predict the changes to RSNA that can be expected with changes in pressure. The infusion of phenylephrine (0.06 mg/ml concentration) was increased at 1-min intervals from 50 to 100, 200, 300 and 400 ml/hr using an infusion pump.

At the end of the study, all the animals were euthanized with i.v. infusion of sodium pentobarbital.

### Data analysis

The experimenters on the day were responsible for carrying out the embolization protocol and therefore were not blinded to the groups. The data analysis was conducted by the same researchers, so the analysis was not blinded. Both systolic (SBP) and diastolic (DBP) pressure, RBF, RSNA and ECG were recorded using a data acquisition unit (Micro 1401-3, Cambridge Electronic Design, UK) and data acquisition software (Spike2, Cambridge Electronic Design, UK). Other parameters were calculated during offline data analysis [i.e., mean arterial pressure (MAP), pulse pressure (PP = SBP − DBP), RSNA (number of spikes crossing the threshold and averaged for the heart interval duration), and renal resistive index (RRI); (maximum flow − minimum flow/maximum flow)]. Flow was used as measured directly by the flow probes to calculate the RRI. The number of discriminated spikes above threshold between diastolic pressures and corrected for heart period was used as a measure of RSNA such that the measure was in spikes/s. The threshold was set just above background so that spikes from small bursts were counted. The background noise was taken as the spikes per second during the highest dose of phenylephrine when RSNA was abolished, and this was subtracted from the data collected. Two minutes of continuous data were averaged at each stage of cardiac pump support. Although we initiated experiments on 10 sheep for the AMI group, two sheep died before completion of the experimental protocol, enabling us to use only eight sheep for the AMI group. Of those eight sheep, we could not record clear RSNA in one sheep, and another did not have adequate RBF waveforms; thus they were excluded in the analysis for each component. Recordings from seven sheep in the AMI group were used for RSNA and RBF analysis.

In both the AMI and control groups, *n* = 6 were included in the analysis of baroreflex curves. Baseline RSNA 30 min after the coronary artery embolization immediately prior to phenylephrine infusion was used to calculate the 100% RSNA activity. In the controls, the RSNA before the start of the phenylephrine infusion was used as 100%. DBP changes were sorted into 2 mmHg bins in Excel from baseline to the maximum change in DBP, and the mean RSNA of each bin was calculated. The change in RSNA from baseline was calculated as a percentage and expressed against the change in DBP from baseline.

### Statistical analysis

Samples sizes were determined using power calculations (significance criterion of *p* < 0.05 and power of 80%). Using our specific criteria for change in variables, the minimum sample size of *n* = 6 gave us the ability to detect significant changes of ~10% (MAP), 10% (PP), 15% (RSNA) and 10% (RBF). Data are presented as the mean ± standard error of the mean (SEM). Statistical tests were conducted in SPSS (IBM, version 28). GraphPad Prism version 9.3.1 (GraphPad Software Inc., San Diego, CA, USA) was used for generating graphs. Once data were tested for normality, one-way ANOVA or paired two-tailed *t*-test was used to compare means between pump levels/time points, and unpaired two-tailed *t*-test when values between groups were compared.

## Results

### Effect of left coronary artery embolization

The haemodynamic parameters and the renal flow parameters at baseline are presented in Table 1. The two groups were comparable in MAP, DBP, SBP, PP and renal flow parameters (*p* > 0.05) at the start of the protocol before embolization. Injection of microspheres into the coronary artery resulted in a change in the ST segment as visualized in the ECG trace (Fig. 2). When examined at the 30-min mark post-embolization, DBP, SBP, MAP and PP decreased by 16.8 ± 4.1 mmHg, 20.8 ± 4.4 mmHg, 19.1 ± 3.8 mmHg and 2.7 ± 0.1 mmHg, respectively. MAP, SBP and DBP were significantly reduced following embolization (one-way ANOVA; *p* < 0.05). There was a steady increase in RSNA over time, so that at the 30-min point, RSNA was increased by 67.5 ± 25.5% and RBF reduced by 65.2 ± 17.5 ml/min (Fig. 2). RRI increased from 0.71 ± 0.15 at baseline to 1.12 ± 0.41 at 30 min post-embolization. Compared to the controls, the DBP, SBP and MAP measurements were significantly lower in the AMI group after embolization (*p* < 0.05) (Table 1).

**Table 1:** Baseline values of arterial pressure and renal flow in control and AMI groups

Parameters	Control		AMI	
		Pre-embolization	Post-embolization 30 min	Pre-pump
	(*n* = 6)		(*n* = 8)	
HR	99.13 ± 4.48	91.35 ± 4.92	93.27 ± 4.34	90.02 ± 6.59
Blood pressure parameters				
	(*n* = 6)		( n = 8)	
DBP (mmHg)	83.53 ± 8.46^§^	78.08 ± 3.69	61.30 ± 4.90*	48.46 ± 16.00*
SBP (mmHg)	109.34 ± 9.54^§^	104.10 ± 4.75	83.33 ± 4.90*	70.33 ± 17.64*
MAP (mmHg)	92.03 ± 8.77^§^	86.87 ± 4.06	67.77 ± 4.40*	55.69 ± 16.44*
PP (mmHg)	24.15 ± 2.61	25.64 ± 1.82	21.68 ± 1.16	21.86 ± 4.46
Renal flow parameters				
	Control		AMI	
	(*n* = 6)		(*n* = 7)	
RBF ml/min	191.88 ± 69.88	239.56 ± 44.94	220.18 ± 41.83	202.05 ± 69.54
RRI	0.51 ± 0.07	0.72 ± 0.15	1.12 ± 0.41	0.81 ± 0.19

*HR* heart rate, *DBP* diastolic blood pressure, *SBP* systolic blood pressure, *MAP* mean arterial pressure, *PP* pulse pressure, *RBF* renal blood flow, *RRI* renal resistive index

* Pair-wise comparison with pre-embolization values, *p* < 0. 05

^§^Independent-samples two-tailed *t*-test comparing controls with post-embolization values, *p* < 0. 05

### Effect of Impella left ventricular support in AMI

**Fig. 3:**
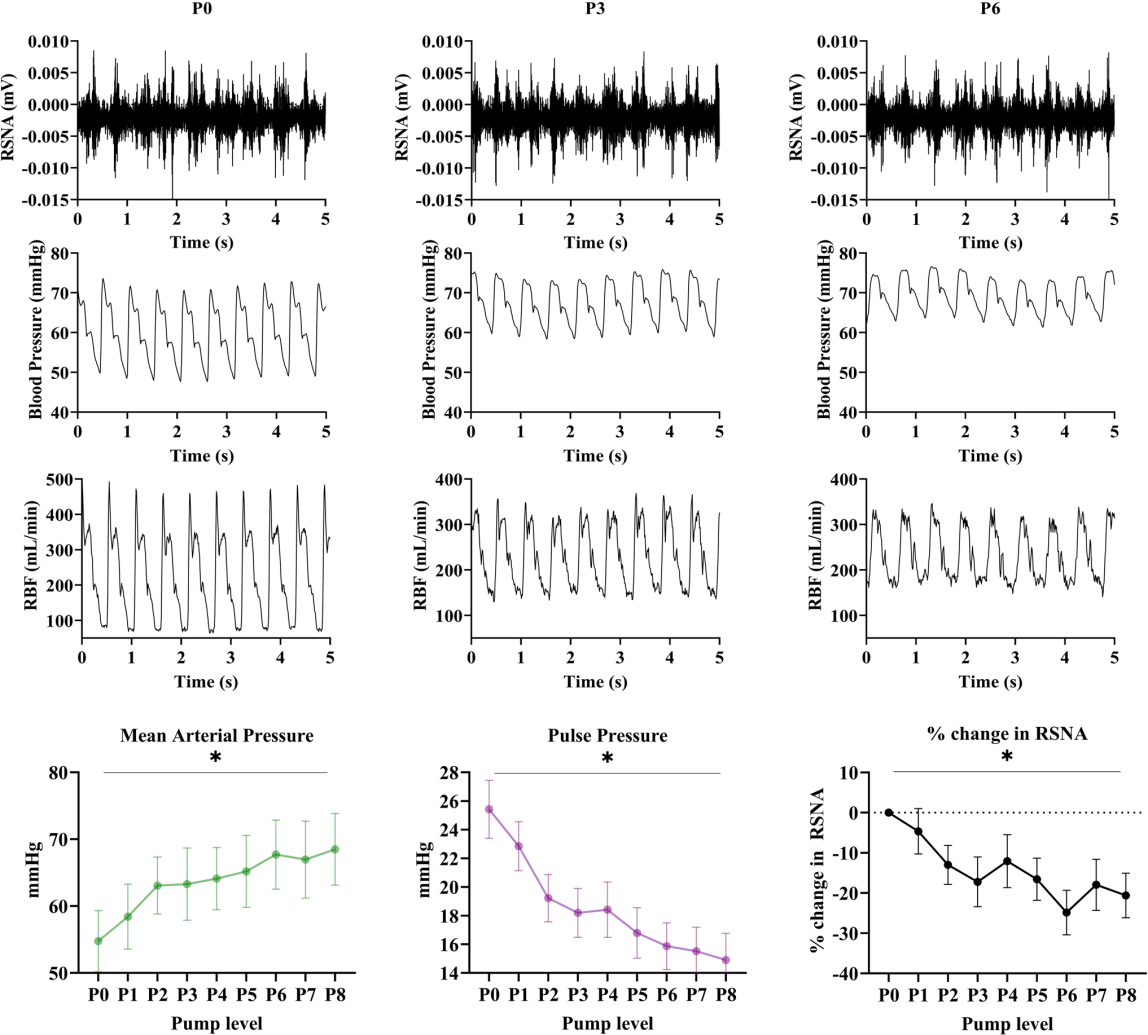
Representative raw data traces of renal sympathetic nerve activity (RSNA), blood pressure and renal blood flow (RBF) at P0, P3 and P6 Impella CP cardiac pump levels in a sheep during AMI-induced left ventricular systolic dysfunction. P0 was recorded by pump insertion into the left ventricle without turning the pump on. Bottom row shows the mean ± SEM of mean arterial pressure (MAP), pulse pressure (PP) and % change in RSNA from P0 (**n** = 8) at incremental pump support in AMI group. * *p* < 0.05 on ANOVA for pump levels

**Fig. 4:**
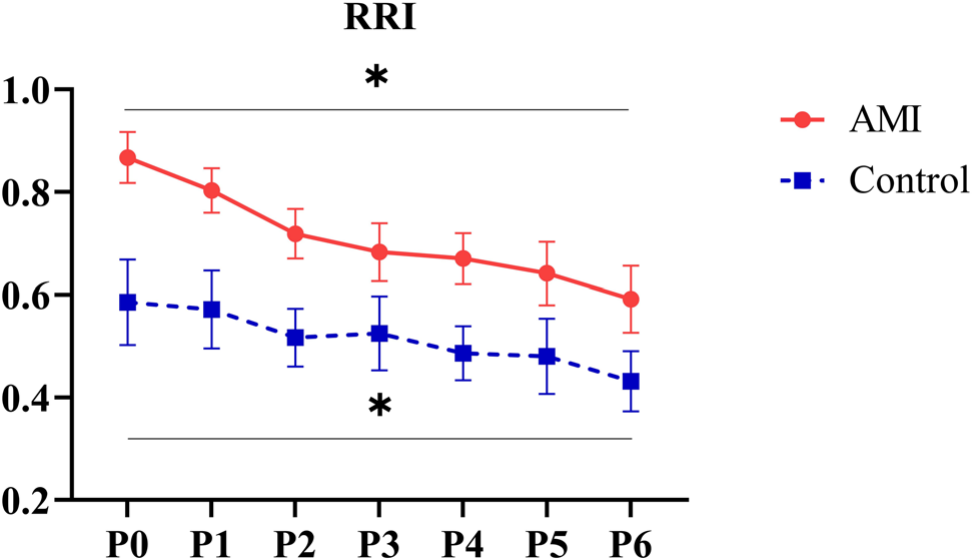
Renal resistive index (RRI) in AMI (**n** = 7) and control (**n** = 6) groups. * *p* < 0.05 on ANOVA for pump levels in both groups. The RRI in the AMI group increased after embolization, then reduced with incremental pump support. In controls, RRI decreased with pump support similar to the AMI group

The Impella circulatory support pump flow rate increased with increasing pump speeds. This support significantly increased MAP from 54.7 ± 4.6 mmHg to 68.5 ± 5.2 mmHg at pump level P8 (one-way ANOVA P0–P6, *p* < 0.001). Incremental pump support in the AMI group (*n* = 7) resulted in a significant decrease in RSNA (one-way ANOVA; *p* < 0.001), with a 20.6 ± 5.5% reduction in RSNA at P8 compared to P0. RBF was improved by 32.6 ± 12.3 ml/min (*n* = 7) at P8 compared to P0 (Fig. 3 and Table 2). There was a decrease in RRI with progressive ventricular support (one-way ANOVA, *p* < 0.001), with a reduction of 0.27 ± 0.05 from P0 to P8 (Fig. 4 and Table 2). The % change in RSNA showed a significant positive correlation with the RRI (*r* = 0.30, *p* = 0.037). To determine whether the change in RSNA was sustained, we recorded RSNA levels for a further 20 min, and this decrease in RSNA continued to remain decreased and was sustained at 20 min of P8 support (−18.6 ± 5.7%, one-way ANOVA; *p* < 0.001;*n* = 3). Impella support in the control animals had a similar effect on all variables from P0 to P6 (Table 2).

**Table 2:** Absolute change in haemodynamic variables and RSNA with incremental Impella support in control and AMI groups

Parameter	Pump level	Control(*n* = 6)	AMI (*n* = 8)
Δ HR (bpm)			
	P2	2.40 ± 2.32	0.64 ± 0.70
	P4	0.04 ± 1.28	−0.92 ± 0.96
	P6	1.92 ± 1.98	−3.01 ± 2.58
	P8		−4.98 ± 3.56
Δ DBP (mmHg)		*	*
	P2	10.17 ± 5.98	12.00 ± 2.22^¶^
	P4	11.34 ± 3.61^¶^	13.77 ± 2.81^¶^
	P6	15.96 ± 5.61^¶^	19.05 ± 4.16^¶^
	P8		19.64 ± 3.50^¶^
Δ SBP (mmHg)			*
	P2	5.01 ± 5.31	3.81 ± 1.56
	P4	3.20 ± 2.44	4.45 ± 1.75
	P6	6.11 ± 4.56	6.00 ± 1.82
	P8		6.00 ± 2.05^¶^
Δ MAP (mmHg)			*
	P2	8.62 ± 5.72	8.10 ± 1.04^¶^
	P4	8.79 ± 3.13	9.18 ± 1.29^¶^
	P6	12.87 ± 5.22	12.52 ± 1.47^¶^
	P8		13.43 ± 1.52^¶^
Δ PP (mmHg)		*	*
	P2	−5.12 ± 1.64^¶^	−6.21 ± 0.89^¶^
	P4	−8.21 ± 2.26^¶^	−7.01 ± 0.71^¶^
	P6	−9.86 ± 2.10^¶^	−9.55 ± 0.95^¶^
	P8		−10.51 ± 1.13^¶^
Δ RBF (ml/min)			
	P2	7.75 ± 10.05	21.34 ± 7.93
	P4	6.10 ± 8.12	29.68 ± 9.58
	P6	16.26 ± 15.35	45.73 ± 20.10
	P8		32.57 ± 12.30
Δ RRI		*	*
	P2	−0.07 ± 0.04^¶^	−0.14 ± 0.01^¶^
	P4	−0.10 ± 0.04^¶^	−0.20 ± 0.03^¶^
	P6	−0.15 ± 0.04^¶^	−0.27 ± 0.04^¶^
	P8		−0.27 ± 0.05^¶^
Δ RSNA (%)		*	*
	P2	−11.33 ± 4.62^¶^	−13.00 ± 4.87^¶^
	P4	−14.99 ± 4.87^¶^	−12.06 ± 6.59^¶^
	P6	−13.25 ± 5.54^¶^	−24.86 ± 5.53 ^¶^
	P8		−20.60 ± 5.52 ^¶^

HR heart rate, *DBP* diastolic blood pressure, *SBP* systolic blood pressure, *MAP* mean arterial pressure, *PP* pulse pressure, *RBF* renal blood flow, *RRI* renal resistive index, *RSNA* renal sympathetic nerve activity

* ANOVA for all pump levels < 0. 05

^¶^Post hoc pair-wise comparison with P0 (following ANOVA), *p* < 0.05

### Effect of pressure changes on RSNA

**Fig. 5:**
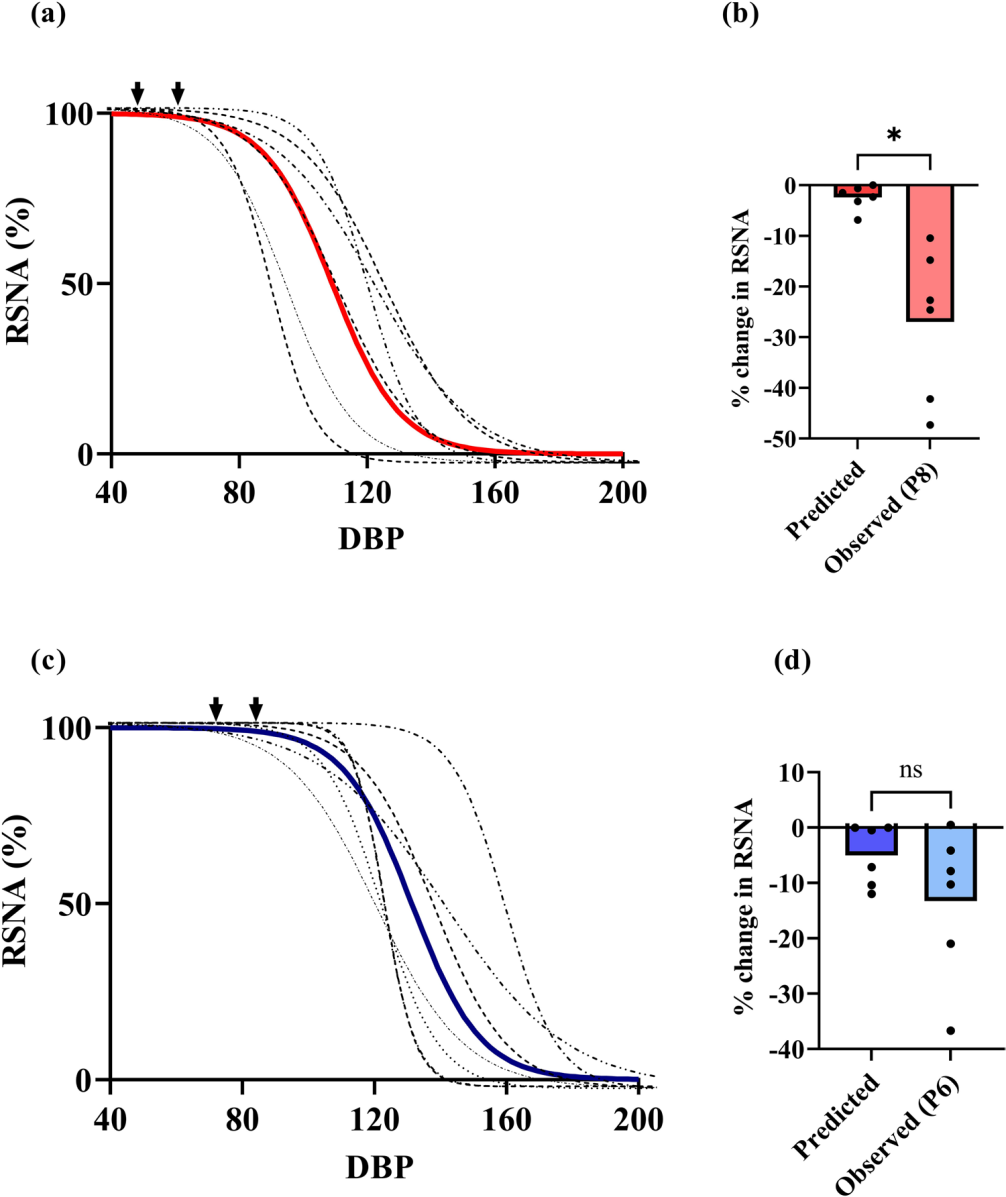
**a** Renal sympathetic nerve activity (RSNA) baroreflex function curves in the AMI group (before pump insertion; **n** = 8). Diastolic blood pressure (DBP change) from P0 to P8 is depicted by arrows. **b** Observed and predicted RSNA % change (using the baro curves) at P8 in the AMI group (*p = 0.006, on paired-samples test). **c** RSNA baroreflex function curves for control sheep (before pump insertion; **n** = 6). Diastolic blood pressure (DBP change) from P0 to P6 is depicted by arrows. **d** Observed and predicted RSNA % change (using the baro curves) at P6 (*ns* **p** > 0.05, on paired-samples test)

Infusion of phenylephrine resulted in an increase in MAP and a reduction in RSNA. When the baro curves were used to predict the changes to RSNA with pressure increases (using phenylephrine), only a 2.6 ± 1.0% decrease in RSNA at P8 (*n* = 6) equivalent pressures was expected in AMI (Fig. 5). The expected change in RSNA in each animal was compared to the observed RSNA change (22.2 ± 5.5%; *n* = 6) with the Impella pump. Interestingly, the decrease in RSNA with the Impella pump was higher than the decrease in RSNA with an increase in blood pressure with phenylephrine (*p* = 0.017). In the control group, the decrease in RSNA with the phenylephrine-induced change was 5.0 ± 2.2%, which was not significantly different from the actual reduction seen with the Impella pump at P6 (13.2 ± 5.5%; *p* = 0.065). In the control group, the decrease in RSNA did not appear to be dependent on the changes in pulse pressure.

**Fig. 6:**
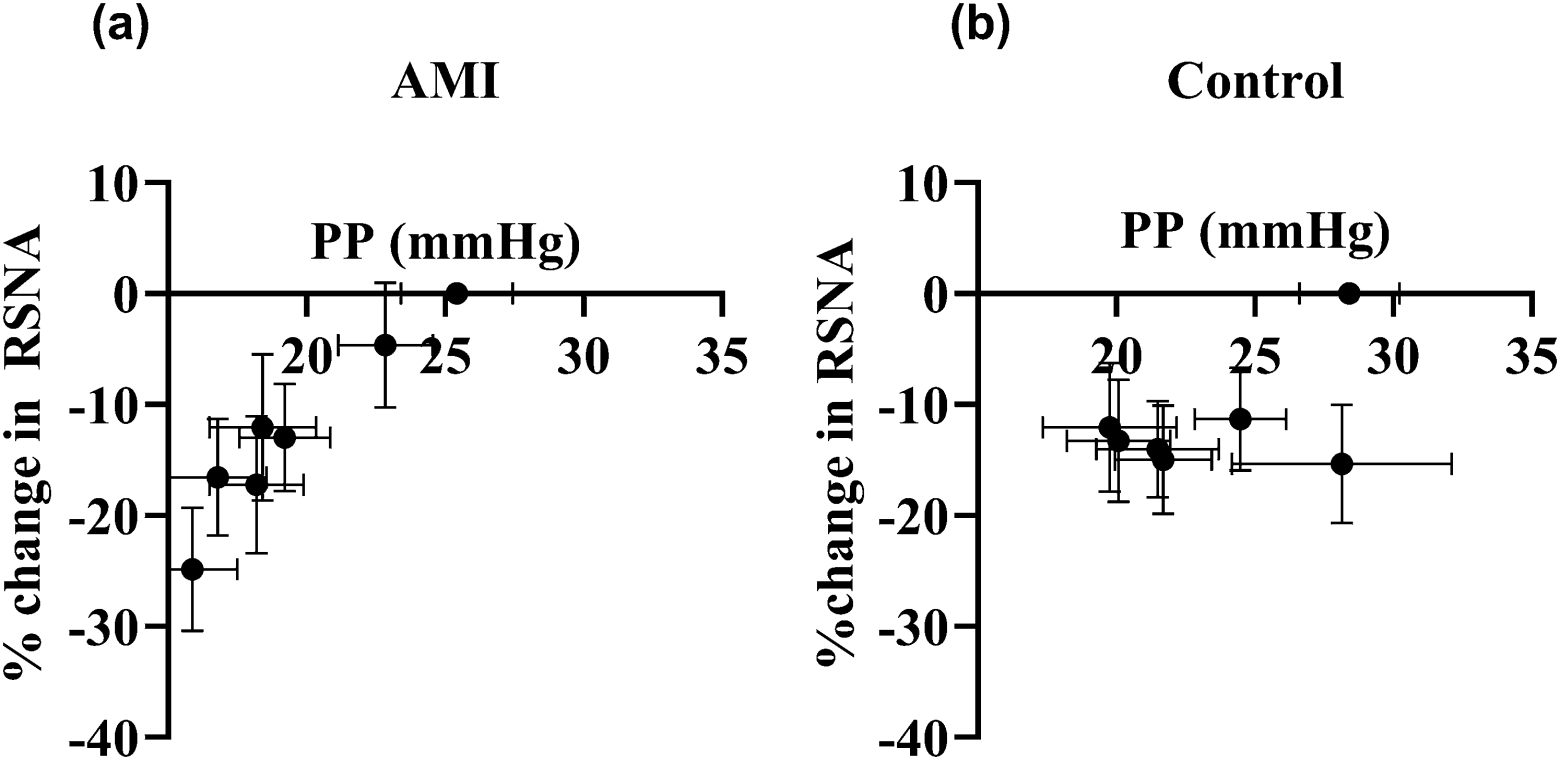
The effect of pulse pressure (PP) changes on renal sympathetic nerve activity (RSNA). **a** In AMI (**n** = 7), the PP was lower with incremental pump support, but the RSNA was lower at these high pump levels. **b** In controls (**n** = 6), although the PP was lower with incremental pump support, RSNA did not show a significant change

The pulse pressure was reduced with increasing pump support in the AMI group (Fig. 3), and we plotted the change in RSNA against this change in PP. Although the pulse pressure was reduced at progressively higher pump levels, the RSNA decreased in parallel in the AMI group (Fig. 6). In contrast, in the control group, the reduction in RSNA appeared to be a step decrease with no further reductions as PP decreased (Fig. 6).

## Discussion

To the best of our knowledge, this is the first study to examine the effects of the Impella LVAD on directly recorded RSNA. The main findings of our study are as follows: (1) RSNA decreased with incremental pump support both in the AMI group and in control animals. During AMI-induced LVSD, a 21% reduction in RSNA was noted with maximum pump support (P8) compared to no-pump support (P0). (2) There was a progressive decline in RRI with Impella pump support in the AMI group as well as the control group. (3) The decrease in RSNA with incremental Impella pump support in the AMI group was significantly greater than that predicted by a pharmacologically induced increase in arterial pressure alone. In contrast, we did not find a difference in the control group.

### Changes in sympathetic nerve activity with the Impella LVAD

RSNA decreased with incremental pump support in both control animals and the AMI group. Since Impella support also increased MAP, we hypothesized that the reduction in renal sympathetic drive would be mediated by the arterial baroreflex [18]. In this context, Tank et al. examined patients with LVADs and found that LVAD support was associated with a reduction in muscle sympathetic nerve activity. Their observation that increased DBP with incremental pump speed resulted in lower muscle sympathetic nerve activity suggested an arterial baroreflex mechanism, although this was not directly tested [19].

We tested the reductions in RSNA during an increase in MAP versus the reduction in RSNA using Impella support in our study. Interestingly, the observed suppression of RSNA with pump support was significantly greater than that predicted by the baroreflex curves at equivalent pressures in the AMI group (Fig. 5). This suggested that the decrease in RSNA could not be solely pressure-mediated and led us to conclude that mechanisms other than the baroreflex may be responsible for the reduction in RSNA. Potential mediators include non-baroreceptor afferent signals from the heart and the kidney. The improvement in ventricular support reduces wall strain on the left ventricle [20–22], improving coronary flow and reducing myocardial ischaemia [23–25]. It is known that ischaemia can increase cardiac afferent activity [26, 27], and this would theoretically be reduced with pump support. Furthermore, it is possible that pulmonary congestion is eased by the pump, and the pulmonary pressures may also decrease [23, 28], leading to less activation of the afferents in the pulmonary circulation. Finally, improved perfusion of the kidney will mean less activation of renal afferents [29], which via the reno-renal reflex may lead to reduced sympathetic activation. We hypothesize that all these mechanisms operated in the AMI group compared to the controls, although we acknowledge that this was not directly tested.

### Renal protective effect of Impella LVAD

AKI is a common but serious complication following AMI [7, 30], and the use of mechanical circulatory assist devices have been shown to reduce in-hospital incidence of AKI and the need for haemodialysis by improving GFR and lowering creatinine levels significantly [10, 13]. Renal perfusion as measured by RRI is known to improve with the use of Impella support [31], and this was again confirmed in our study. Here we demonstrate that the change in RSNA parallels the change in RRI in AMI, suggesting that at least part of the reno-protective effect of Impella in AMI-induced LVSD may be via renal sympathoinhibition. This is an important consideration, since the kidneys receive 17% of the CO in sheep [32] and ~20% in humans [33], the modulation of which will affect cardiac strain. The tendency for peak renal flow during systole to be diminished while diastolic blood flow was significantly elevated resulted in stable RBF measurement consistent with the decrease in PP. It is also possible that improvements in diastolic blood flow to the kidney alone, in the absence of RSNA changes, are enough to improve renal function, although this remains an untested hypothesis at present.

### Pulse pressure effects on RSNA

Pulsatility in blood pressure is important to promote reflex inhibition of sympathetic nerve activity [34–36]. In this context, continuous-flow LVADs caused an increase in muscle sympathetic nerve activity at incremental pump speeds [37], and heart failure patients on continuous-flow LVADs reported higher SNA when compared to patients on pulsatile-flow LVADs [38]. To determine whether further reductions in pulse pressure during Impella use may alter the inhibition of RSNA, we plotted the change in RSNA with the change in PP. In our study, there was a progressive decrease in both PP and RSNA with incremental pump support in the AMI group (Fig. 6), indicating that while PP was diminished, there was enough residual PP with pump support to alter RSNA. The observation that the reduction in PP with incremental pump speed resulted in a reduction in muscle sympathetic nerve activity in patients with heart failure [19] suggests that the reduction in RSNA appears to be despite the putative decreases in baro-afferent discharge that would be associated with the reduction in PP. We will note that while there was no association with decreases in RSNA and PP in the control group, the PP did not drop below 20 mmHg, which may be one reason for not observing a relationship.

### Study limitations

We were unable to measure urine output or other urinary measures of kidney function to confirm that the reduction in RSNA was associated with improvements in kidney function, although previous clinical studies have shown improvements in kidney function with Impella use [11, 12]. We acknowledge that the use of phenylephrine to assess the baroreflex is not comparable to pharmacological management of AMI-induced hypotension and impending cardiogenic shock. These experiments were conducted under anaesthesia, and isoflurane anaesthesia is known to increase levels of RSNA in sheep [40, 41]. Long-term changes to RSNA were not evaluated in the current study, and it is possible that with long-term use, vascular modifications in both the major arteries and the renal vasculature [42] might alter SNA. The long-term effects of Impella use on RSNA and kidney function remain to be determined.

## Conclusions

These data suggest that unloading of the heart using Impella may have a greater inhibitory effect on renal sympathetic drive than maintaining blood pressure with the use of vasopressor agents. Taken together, these data suggest a mechanistic pathway whereby unloading of the left ventricle delays progression of AKI in AMI.

## Data Availability

Data used in the current study is available upon reasonable request.

## References

[1] World Health Organization. Global Health Observatory data repository. 2021. https://www.who.int/data/gho/data/themes/mortality-and-global-health-estimates/ghe-leading-causes-of-death.

[2] Liang JJ, Fender EA, Cha Y-M, Lennon RJ, Prasad A, Barsness GW (2016) Long-term outcomes in survivors of early ventricular arrhythmias after acute ST-elevation and Non–ST-elevation myocardial infarction treated with percutaneous coronary intervention. Am J Cardiol 117:709–713. 10.1016/j.amjcard.2015.12.002 26796195 10.1016/j.amjcard.2015.12.002

[3] Basir MB, Schreiber T, Dixon S, Alaswad K, Patel K, Almany S et al (2018) Feasibility of early mechanical circulatory support in acute myocardial infarction complicated by cardiogenic shock: the detroit cardiogenic shock initiative. Catheter Cardiovasc Interv 91:454–461. 10.1002/ccd.27427 29266676 10.1002/ccd.27427

[4] Sjauw KD, Remmelink M, Baan J, Lam K, Engström AE, van der Schaaf RJ et al (2008) Left ventricular unloading in acute ST-segment elevation myocardial infarction patients is safe and feasible and provides acute and sustained left ventricular recovery. J Am Coll Cardiol 51:1044–1046. 10.1016/j.jacc.2007.10.050 18325447 10.1016/j.jacc.2007.10.050

[5] McDonagh TA, Metra M, Adamo M, Gardner RS, Baumbach A, Böhm M et al (2021) 2021 ESC guidelines for the diagnosis and treatment of acute and chronic heart failure. Eur Heart J 42:3599–3726. 10.1093/eurheartj/ehab368 34447992 10.1093/eurheartj/ehab368

[6] Heidenreich PA, Bozkurt B, Aguilar D, Allen LA, Byun JJ, Colvin MM et al (2022) AHA/ACC/HFSA guideline for the management of heart failure: a report of the American college of cardiology/American heart association joint committee on clinical practice guidelines. Circulation 2022:145. 10.1161/CIR.0000000000001063 10.1161/CIR.0000000000001063 35363499

[7] Cosentino N, Resta ML, Somaschini A, Campodonico J, Lucci C, Moltrasio M et al (2021) Acute kidney injury and in-hospital mortality in patients with ST-elevation myocardial infarction of different age groups. Int J Cardiol 344:8–12. 10.1016/j.ijcard.2021.09.023 34537309 10.1016/j.ijcard.2021.09.023

[8] Hutton I, Lindsay RM, Pack AI, Lawrie TDV (1970) Clinical significance of renal haemodynamics in acute myocardial infarction. Lancet 296:123–125. 10.1016/S0140-6736(70)92702-9 10.1016/s0140-6736(70)92702-9 4194504

[9] Kaltsas E, Chalikias G, Tziakas D (2018) The incidence and the prognostic impact of acute kidney injury in acute myocardial infarction patients: current preventive strategies. Cardiovasc Drugs Ther 32:81–98. 10.1007/s10557-017-6766-6 29349645 10.1007/s10557-017-6766-6

[10] Flaherty MP, Pant S, Patel SV, Kilgore T, Dassanayaka S, Loughran JH et al (2017) Hemodynamic support with a microaxial percutaneous left ventricular assist device (Impella) protects against acute kidney injury in patients undergoing high-risk percutaneous coronary intervention. Circ Res 120:692–700. 10.1161/CIRCRESAHA.116.309738 28073804 10.1161/CIRCRESAHA.116.309738

[11] Flaherty MP, Moses JW, Westenfeld R, Palacios I, O’Neill WW, Schreiber TL et al (2020) Impella support and acute kidney injury during high-risk percutaneous coronary intervention: the global cVAD renal protection study. Catheter Cardiovasc Interv 95:1111–1121. 10.1002/ccd.28400 31355987 10.1002/ccd.28400

[12] Patsalis N, Kreutz J, Chatzis G, Syntila S, Griewing S, Pirlet-Grant C et al (2022) Renal protection and hemodynamic improvement by Impella^®^ microaxial pump in patients with cardiogenic shock. J Clin Med 11:6817. 10.3390/jcm11226817 36431294 10.3390/jcm11226817 PMC9698353

[13] Bashline MJ, Rhinehart Z, Kola O, Fowler J, Kaczorowski D, Hickey G (2022) Impella 5.0 is associated with a reduction in vasoactive support and improves hemodynamics in cardiogenic shock: A single-center experience. Int J Artif Organs. 10.1177/03913988221083993 35365048 10.1177/03913988221083993

[14] Osborn JW, Tyshynsky R, Vulchanova L (2021) Function of renal nerves in kidney physiology and pathophysiology. Annu Rev Physiol 83:429–450. 10.1146/annurev-physiol-031620-091656 33566672 10.1146/annurev-physiol-031620-091656

[15] Sohn CS-E, Chang JW-H, George B, Chen S, Ramchandra R (2022) Role of the angiotensin type 1 receptor in modulating the carotid chemoreflex in an ovine model of renovascular hypertension. J Hypertens. 10.1097/HJH.0000000000003173 35762481 10.1097/HJH.0000000000003173

[16] Shanks J, Abukar Y, Lever NA, Pachen M, LeGrice IJ, Crossman DJ et al (2022) Reverse re-modelling chronic heart failure by reinstating heart rate variability. Basic Res Cardiol. 10.1007/s00395-022-00911-0 35103864 10.1007/s00395-022-00911-0 PMC8807455

[17] Watson AMD, Mogulkoc R, McAllen RM, May CN (2004) Stimulation of cardiac sympathetic nerve activity by central angiotensinergic mechanisms in conscious sheep. Am J Physiol Regul Integr Comp Physiol 286:R1051–R1056. 10.1152/ajpregu.00708.2003 14751846 10.1152/ajpregu.00708.2003

[18] DiBona GF, Sawin LL (1994) Reflex regulation of renal nerve activity in cardiac failure. Am J Physiol Integr Comp Physiol 266:R27-39. 10.1152/ajpregu.1994.266.1.R27 10.1152/ajpregu.1994.266.1.R27 8304550

[19] Tank J, Heusser K, Malehsa D, Hegemann K, Haufe S, Brinkmann J et al (2012) Patients with continuous-flow left ventricular assist devices provide insight in human baroreflex physiology. Hypertension 60:849–855. 10.1161/HYPERTENSIONAHA.112.198630 22824986 10.1161/HYPERTENSIONAHA.112.198630

[20] Sauren LDC, Accord RE, Hamzeh K, de Jong M, van der Nagel T, van der Veen FH et al (2007) Combined Impella and intra-aortic balloon pump support to improve both ventricular unloading and coronary blood flow for myocardial recovery: an experimental study. Artif Organs 31:839–842. 10.1111/j.1525-1594.2007.00477.x 18001394 10.1111/j.1525-1594.2007.00477.x

[21] Pal G, Pal P, Nanda N (2017) Principles of Hemodynamics. Comprehensive Textbook of Medical Physiology. Jaypee Brothers Medical Publishers (P) Ltd, Japan

[22] Burkhoff D, Sayer G, Doshi D, Uriel N (2015) Hemodynamics of mechanical circulatory support. J Am Coll Cardiol 66:2663–2674. 10.1016/j.jacc.2015.10.017 26670067 10.1016/j.jacc.2015.10.017

[23] Udesen NLJ, Helgestad OKL, Banke ABS, Frederiksen PH, Josiassen J, Jensen LO et al (2020) Impact of concomitant vasoactive treatment and mechanical left ventricular unloading in a porcine model of profound cardiogenic shock. Crit Care 24:95. 10.1186/s13054-020-2816-8 32188462 10.1186/s13054-020-2816-8 PMC7079533

[24] Fukamachi D, Yamada A, Ohgaku A, Koyama Y, Fujito H, Arai R et al (2022) Protective effect of the Impella on the left ventricular function after acute broad anterior wall ST elevation myocardial infarctions with cardiogenic shock: cardiovascular magnetic resonance imaging strain analysis. BMC Cardiovasc Disord 22:201. 10.1186/s12872-022-02632-7 35484492 10.1186/s12872-022-02632-7 PMC9052554

[25] Watanabe S, Fish K, Kovacic JC, Bikou O, Leonardson L, Nomoto K et al (2018) Left ventricular unloading using an Impella CP improves coronary flow and infarct zone perfusion in ischemic heart failure. J Am Heart Assoc. 10.1161/JAHA.117.006462 29514806 10.1161/JAHA.117.006462 PMC5907535

[26] Staszewska-Barczak J, Dusting GJ (1977) Sympathetic cardiovascular reflex initiated by bradykinin-induced stimulation of cardiac pain receptors in the dog. Clin Exp Pharmacol Physiol 4:443–452. 10.1111/j.1440-1681.1977.tb02408.x 269762 10.1111/j.1440-1681.1977.tb02408.x

[27] Malik R, Minisi AJ (1997) Simultaneous cardiac and renal sympathetic neural responses to activation of left ventricular sympathetic afferents. J Auton Nerv Syst 65:10–16. 10.1016/S0165-1838(97)00029-5 9258867 10.1016/s0165-1838(97)00029-5

[28] Lim HS (2017) The effect of Impella on cardiopulmonary physiology during venoarterial extracorporeal membrane oxygenation support. Artif Organs 41:1109–1112. 10.1111/aor.12923 28591467 10.1111/aor.12923

[29] Ciriello J, de Oliveira CVR (2002) Renal afferents and hypertension. Curr Hypertens Rep 4:136–142. 10.1007/s11906-002-0038-x 11884269 10.1007/s11906-002-0038-x

[30] Singh S, Kanwar A, Sundaragiri PR, Cheungpasitporn W, Truesdell AG, Rab ST et al (2021) Acute kidney injury in cardiogenic shock: an updated narrative review. J Cardiovasc Dev Dis 8:88. 10.3390/jcdd8080088 34436230 10.3390/jcdd8080088 PMC8396972

[31] Markus B, Patsalis N, Chatzis G, Luesebrink U, Ahrens H, Schieffer B et al (2020) Impact of microaxillar mechanical left ventricular support on renal resistive index in patients with cardiogenic shock after myocardial infarction: a pilot trial to predict renal organ dysfunction in cardiogenic shock. Eur Hear J Acute Cardiovasc Care 9:158–163. 10.1177/2048872619860218 10.1177/2048872619860218 PMC7068781 31246097

[32] Upton RN (2008) Organ weights and blood flows of sheep and pig for physiological pharmacokinetic modelling. J Pharmacol Toxicol Methods 58:198–205. 10.1016/j.vascn.2008.08.001 18775498 10.1016/j.vascn.2008.08.001

[33] O’Connor PM (2006) Renal oxygen delivery: Matching delivery to metabolic demand. Clin Exp Pharmacol Physiol 33:961–967. 10.1111/j.1440-1681.2006.04475.x 17002675 10.1111/j.1440-1681.2006.04475.x

[34] Fukae K, Tominaga R, Tokunaga S, Kawachi Y, Imaizumi T, Yasui H (1996) The effects of pulsatile and nonpulsatile systemic perfusion on renal sympathetic nerve activity in anesthetized dogs. J Thorac Cardiovasc Surg 111:478–484. 10.1016/S0022-5223(96)70459-2 8583823 10.1016/s0022-5223(96)70459-2

[35] Angell James JE, de Daly M, B. (1971) Effects of graded pulsatile pressure on the reflex vasomotor responses elicited by changes of mean pressure in the perfused carotid sinus-aortic arch regions of the dog. J Physiol. 10.1113/jphysiol.1971.sp009418 5575376 10.1113/jphysiol.1971.sp009418 PMC1331821

[36] Chapleau MW, Hajduczok G, Abboud FM (1989) Pulsatile activation of baroreceptors causes central facilitation of baroreflex. Am J Physiol Circ Physiol 256:H1735–H1741. 10.1152/ajpheart.1989.256.6.H1735 10.1152/ajpheart.1989.256.6.H1735 2735443

[37] Cornwell WK, Tarumi T, Stickford A, Lawley J, Roberts M, Parker R et al (2015) Restoration of pulsatile flow reduces sympathetic nerve activity among individuals with continuous-flow left ventricular assist devices. Circulation 132:2316–2322. 10.1161/CIRCULATIONAHA.115.017647 26510698 10.1161/CIRCULATIONAHA.115.017647

[38] Markham DW, Fu Q, Palmer MD, Drazner MH, Meyer DM, Bethea BT et al (2013) Sympathetic neural and hemodynamic responses to upright tilt in patients with pulsatile and nonpulsatile left ventricular assist devices. Circ Hear Fail 6:293–299. 10.1161/CIRCHEARTFAILURE.112.969873 10.1161/CIRCHEARTFAILURE.112.969873 23250982

[39] Tarvasmäki T, Haapio M, Mebazaa A, Sionis A, Silva-Cardoso J, Tolppanen H et al (2018) Acute kidney injury in cardiogenic shock: definitions, incidence, haemodynamic alterations, and mortality. Eur J Heart Fail 20:572–581. 10.1002/ejhf.958 28960633 10.1002/ejhf.958

[40] Iguchi N, Kosaka J, Booth LC, Iguchi Y, Evans RG, Bellomo R et al (2019) Renal perfusion, oxygenation, and sympathetic nerve activity during volatile or intravenous general anaesthesia in sheep. Br J Anaesth 122:342–349. 10.1016/j.bja.2018.11.018 30770052 10.1016/j.bja.2018.11.018

[41] Taavo M, Rundgren M, Frykholm P, Larsson A, Franzén S, Vargmar K et al (2021) Role of renal sympathetic nerve activity in volatile anesthesia’s effect on renal excretory function. Funct. 10.1093/function/zqab042 10.1093/function/zqab042 PMC8788708 35330795

[42] Ohnishi H, Itoh T, Nishinaka T, Tatsumi E, Fukuda T, Oshikawa M et al (2002) Morphological changes of the arterial systems in the kidney under prolonged continuous flow left heart bypass. Artif Organs 26:974–979. 10.1046/j.1525-1594.2002.07135.x 12406155 10.1046/j.1525-1594.2002.07135.x

[43] Fallick C, Sobotka PA, Dunlap ME (2011) Sympathetically mediated changes in capacitance. Circ Hear Fail 4:669–675. 10.1161/CIRCHEARTFAILURE.111.961789 10.1161/CIRCHEARTFAILURE.111.961789 21934091

